# In vitro evaluation of the effects of capsaicin on normal and cancerous cells of human cartilage

**DOI:** 10.3906/biy-1804-83

**Published:** 2018-10-25

**Authors:** Nagihan HELVACI, Sabahattin CÖMERTPAY

**Affiliations:** 1 Department of Agricultural Biotechnology, Faculty of Agriculture, Kahramanmaraş Sütçü İmam University , Kahramanmaraş , Turkey; 2 Department of Bioengineering and Sciences, Kahramanmaraş Sütçü İmam University , Kahramanmaraş , Turkey

**Keywords:** Chondrosarcoma, chondrocyte, capsaicin, nonapoptotic, caspase-3, real-time polymerase chain reaction, wound healing, migration assay

## Abstract

Chondrosarcoma is a common form of bone cancer which effects the fibrous connective tissue around a joint. It most commonly develops in legs, arms, shoulder blades, rib cage, and pelvis. Capsaicin is an active bitter compound found in red pepper, the fruit of the species Capsicum annuum, and it has been shown to have a lethal effect on different types of cancer. However, to date, investigation of its effect on human chondrosarcoma cells has remained limited. In the study presented here, we determined IC_50_ values of capsaicin for chondrosarcoma and chondrocyte cells in both fetal bovine serum (FBS)-containing and FBS-deprived media, and no statistically significant difference was found between the cell types. Besides, when the cells were cultured with capsaicin at their determined IC_50_ value for 24 h and their caspase-3 gene expression levels were detected by real-time polymerase chain reaction (RTPCR) and western blotting, it was demonstrated that the caspase-3 protein and mRNA levels were not altered in any cells upon capsaicin exposure, suggesting a caspase-independent pathway for cell death. Migration and invasion abilities of the cancerous cells, on the other hand, were observed to decrease dramatically when the cells were exposed to capsaicin (P < 0.05).

## 1. Introduction

Chondrosarcoma is the second most frequently seen primary malignant bone tumor with poor prognosis, and it can be classified into 3 categories according to nuclear size, matrix alterations, cellularity, and mitosis (Evans et al., 1977). The most distinctive characteristic of grade I chondrosarcoma, the type which accounts for nearly 50% of all cases, is its growth rate. In addition to its slow growth, the cells forming the tumor generally have small nuclei, and their chromatin density is quite high. Grade II and III chondrosarcomas are considered to be aggressive tumors since they tend to progress and metastasize rapidly and therefore display a poor survival rate. Studies reported that patients with grade II tumors show 64% survival rate while only 29% of patients with grade III chondrosarcomas manage to survive for 10 years (Van Meldegem et al., 2014a) . Surgical resection is the main treatment for chondrosarcoma; however, it is often difficult and not always practical for those who developed metastasis (Van Meldegem et al., 2014b) . As a result, more robust therapeutic regimens are required to combat this lethal disease. Recently, application of active compounds isolated from plants, particularly those with a history of medicinal use in many ethnic cultures, has become an attractive area of research especially for its biodiversity and great potential in anticancer therapeutics (Lee et al., 2012).

Phytochemical plants are being investigated in vitro and in vivo for their effects on human health at increasingly higher rates. Among these plants, Capsicum annuum L. is commonly used in medicine, chemistry, and the pharmaceutics industry. The active ingredient in Capsicum annuum L. is capsaicin, a spicy, white, and odorless substance (Surh, 2012; Zık and Erdost, 2002) . Capsaicin has been demonstrated to be effective against several types of cancer such as leukemia (Zhang et al., 2003) , multiple myeloma (Bhutani et al., 2007) , cutaneous cell carcinoma (Hail and Lotan 2002), glioma (Lee et al., 2000), tongue cancer (Ip et al., 2012a), nasopharyngeal carcinoma (Ip et al., 2012b), esophageal carcinoma (Wu et al., 2006) , gastric cancer (Kim et al., 1997), pancreatic cancer (Zhang et al., 2008) , hepatocarcinoma (Jung et al., 2001), colon carcinoma (Kim et al., 2004), nonsmall-cell lung cancer (Brown et al., 2010), breast cancer (Chou et al., 2009), and prostate cancer in vitro (Mori et al., 2006).

Studies conducted to reveal the background of capsaicin’s effect on transformed cells showed that capsaicin leads cells to apoptosis by keeping cells in the G0/G1 phase of their cell cycle (Jin et al., 2014). Apoptosis is generally a self-extinguishing, organized, and programmatic cell death which maintains homeostasis in the organism (Hengartner et al., 1992; Andrew et al., 2001) . The central component of the apoptotic program is the group of endoproteases called caspases (Hampton and Orrenius, 1998). Their activation is cell-specific and they can be classified into two groups as the “initiators” of proteolysis (caspase-2,-8,-10) or “practitioners” (caspase-3,-6,-7) (Büyükgebiz and Caferler, 2001; Budihardjo and Oliver, 1999) . In humans, the caspase-3 molecule is considered to be one of the most important caspases whose certain genotypes have been related to the risk of some cancer types such as squamous cell carcinomas of the head and neck (McIlwain et al., 2013).

A cell cycle is a highly regulated process at the end of which a cell is divided and turned into two cells through mitosis. Cell division cycle can be divided into two main phases as the mitotic phase and the interphase. The interphase can be seen through the G1, G2, and S phases. Progression from one phase to another is carried out by the activity of cyclin-dependent kinases which are tightly regulated by the presence of cyclins (Malumbres, 2014). Arrest of the cells in one phase of the cycle can be followed and proven by the sudden decrease in the appropriate cyclin such that the disappearance of cyclin E, an intermediate protein taking a role in progression from the G1 phase to the S phase, can be used as a sign of G1 arrest in cells (Joachim et al., 1996).

Taking all the facts presented above into consideration, we aimed to understand how normal and cancerous cells of cartilage would be affected by in vitro application of capsaicin. The effect was examined via cytotoxicity revealed by MTS Assay, and its apoptotic potential was investigated by determining the caspase-3 levels through western blotting and qRT-PCR. Additionally, the change in the cytotoxicity of capsaicin when the cells’ cyclin E levels were reduced (by growing them in FBS-deprived medium) was also evaluated. Lastly, in cancer cells, we assessed the variation in the invasive capacity of the cells upon capsaicin exposure by using wound healing assay and invasion assay.

## 2. Materials and methods

### 2.1. Cell culture

Human cartilage chondrocyte (CHO) primary cells and Human Chondrosarcoma (OUMS) cell line were obtained from Okayama University Medical School, Dental and Pharmaceutical Sciences Institute in Japan. The cells were grown in M199 (Medium 199; GIBCO®, Invitrogen, Thermo Fisher Scientific, Waltham, MA, USA), supplemented with 10% fetal bovine serum (FBS; GIBCO®, Invitrogen), and 100 units/mL of penicillin and streptomycin (GIBCO®, Invitrogen). They were grown in an incubator (Panasonic, Gunma, Japan) providing a 5%-CO2-containing humidified atmosphere at 37 °C. The cells were passaged by trypsinization once they reached 75%–80% confluence.

### 2.2. Lowered cyclin E levels

The effect of cell cycle arrest in the G1 phase on capsaicintreated cells was assessed by starving the cells for 24 h in serum-free (FBS-deprived) medium. The cells were counted and cultured in flasks with FBS-containing media. Upon their attachment, nearly 16 h later, the FBS-containing medium was replaced with the FBS-free medium in one flask while the control flask was refreshed with the FBS-containing medium. After 24 h, the pellets were collected from both flasks and the proteins were isolated. A subsequent western blotting was performed to measure the cyclin E levels.

### 2.3. Cell viability assay (MTS)

The CHO and OUMS cells were plated on 96-well plates as 5 × 10^3^ cells per well. In order to reveal the dose–response relationship, the cells were treated with capsaicin at a concentration range of 0–600 μM for 24 h. Cell viability was measured by MTS assay (CellTiter 96® AQueous Assay; Promega, Fitchburg, WI, USA) using a spectrophotometer (Spektramax; BMG Labtech., Ofenburg, Germany) at a wavelength of 490 nm. The wells in which only the medium was present were regarded as blank. Measurements were made by three independent experiments (n = 3) where three replicates were read for each condition. After these steps, the IC_50_ values of capsaicin for chondrosarcoma and normal cartilage cells were calculated using GraphPad Prism 6.0 (La Jolla, CA, USA). Capsaicin was applied to the cells at these IC_50_ concentrations to assess its effects in further experiments.

### 2.4. Western blotting

Total protein extraction was performed by using MPER (Thermo Scientific, Rockford, IL, USA) on the cell pellets obtained through trypsinization and subsequent centrifuging. Protein concentrations were determined by Bradford analysis. Proteins (30 µg per lane) were loaded into 12% SDS-PAGE. They were then transferred to a PVDF membrane by electrophoresis. After removal of the PVDF membrane from the transfer system, it was incubated with the blocking solution (5% (w/v)) and skimmed milk powder in 0.1% Tween 20 containing Tris buffered saline (TBST) for 30 min at room temperature. Later, the membrane was treated with the primary antibody specific to the targeted protein (caspase-3, cyclin E, and GAPDH) diluted as 1 in 500 blocking buffer at +4 °C overnight. On the next day, the membrane was washed 5 times with TBST in a shaker and incubated with secondary antibody (antirabbit for caspase-3 and cyclin E; antigoat for GAPDH) diluted as 1 in 500 blocking buffer for 1 h at room temperature. After incubation, the cells were again washed 5 times with TBST in a shaker, and protein bands were visualized under a UVP imaging system (Cambridge, UK) in dark following a short treatment with ECL substrate (BioRad, Hercules, CA, USA). The housekeeping protein, GAPDH, was used for normalization purposes. Normalization was performed by dividing the band intensity of the targeted protein to that of GAPDH in the same well. The band intensities were detected by using the ImageJ software (Bethesda, MD, USA).

### 2.5. Real-time PCR (RT-PCR)

Total RNA extraction from cell pellets was performed using a kit (Vivantis GF-1, Vivantis Technologies, Subang Jaya, Malaysia). The RNA concentration was adjusted to the appropriate amount of RNA for this concentration and the complementary DNA (cDNA) was synthesized with a reverse transcriptase kit (New England BioLabs, Beverly, MA, USA). Quantitative real-time PCR was performed using a real-time PCR machine (Light Cycler Nano, Roche, Mannheim, Germany) and a master mix containing SYBR Green (ABM, Richmond, Canada). The reaction conditions consisted of incubation at 95 °C for 10 min and 45 cycles of 95 °C for 15 s and 60 °C for 60 s. The emission of SYBR Green was read and recorded at the end of each cycle. The primers in Table were used to amplify target genes. While caspase-3 primers were used as given by Lacelle et al. (2002), β-Actin primers were designed by us. In order to design them, we first checked all transcript variants of Homo sapiens Beta-actin gene and copied their sequences to Clone Manager Software (Denver, CO, USA) to align. Of the aligned sequences, the appropriate areas were picked as forward and reverse primers. Prior to running the primers for RT-PCR, a gradient PCR with cDNAs were run to determine the best annealing temperature. After each experiment of RT-PCR, the products were run through a gel to check the amplicon size and purity. Only the results with satisfying clarity were used for further analysis. The expression rates of caspase-3 were determined by normalizing its Ct values to that of β-actin. All ratios of caspase-3/β-actin were compared to the control group whose expression level was set as “1”, and the results are given as relative expression.

**Table 1:** Base sequence of primers used in RT-PCR.

Primer	Base sequence
Human caspase-3	Forward (5’AGAGGGGATCGTTGTAGAAG-3’) Reverse (5’GTTGCCACCTTTCGGTTAAC-3’)
Human β-actin	Forward (5’CCCTGGACTTCGAGCAAGAG-3’) Reverse (5’GATCTTCATTGTGCTGGGTGC-3’)

### 2.6. Wound healing assay

The cells were counted under a microscope with a BurkerTurk lam and then 145,500 cells were seeded in each well of a 6-well plate. After approximately 16 h of waiting for the cells to cling to the surface, the cells were expected to reach approximately 90% confluence. After almost the entire surface was covered with cells, the medium was aspirated from the wells and the surface of the cells was drawn with a sterile micropipette tip. After the scouring, media containing capsaicin, ethanol, or no supplement was placed on the cells and their initial photographs were collected using at least four different sites per well using the JuLI™ Br live imaging system (NanoEnTek Inc., Seoul, Korea). The picture taking process was repeated at 24-h intervals until the scratch formed by the pipette tip was completely closed in at least one of the groups in the experiment. When the experiment was stopped, the photographs were analyzed using the software of the JuLI™ Br live imaging system (NanoEnTek Inc.) and the change in cell density was analyzed via GraphPad Prism 6.0. For the experiments where the FBS-deprived media were used, the media were changed to the ones without FBS once the cell attachment was complete and FBS-deprived media were used in all subsequent treatments.

### 2.7. Migration assay

An invasion assay kit was used according to the manufacturer’s protocol. Briefly, chondrosarcoma cells were seeded into a Boyden chamber (Costar430166, Corning, NY, USA) at a density of 14.6 × 10^3^ cells/per chamber and incubated at 37 °C for 16 h. Serum-free media were added to the upper chamber of a trans-well insert. The bottom well contained a growth medium with 10% FBS. The capsaicin concentration that was used in this assay was different for upper and lower chambers. The upper chamber consisted of capsaicin at the IC_50_ value determined in the FBS-deprived medium while the lower one was supplemented with it at the IC_50_ value calculated for the cells grown in the FBS-containing medium. After 16 h of inculation, the cells treated with or without capsaicin or with ethanol only were washed twice with a phosphate buffer solution (PBS), followed by fixation of 4% paraformaldehyde (PFA) for 5 min. Following the fixation, a crystal violet solution was applied to the cells for 2 min. The chambers were then washed twice with PBS. The images displaying the transferred cells were taken using the JuLI™ Br imaging system (NanoEnTek Inc.). The pictures were analyzed by the ImageJ software.

### 2.8. Data analysis

Statistical analysis was carried out by using GraphPad Prism 6.0. The level of significance between different treatment groups relative to control was determined by Student’s t-test for between-group comparison. P < 0.05 was considered to be statistically significant. All data are presented as mean ± the standard deviation (SD) of three independent experiments. To analyze the difference between the IC_50_ values of the cell types, absorbance values were turned into percentages with the assumption that at a 0 concentration of capsaicin, 100% of the cells were alive. Once the values were adjusted, two-way ANOVA was applied and both the column factor (cell types) and interaction values were considered for the assessment of statistical significance.

## 3. Results

### 3.1. Cancerous and normal cartilage cell viabilities are affected by capsaicin similarly in both FBS-containing and FBS-deprived media

**Figure 1:**
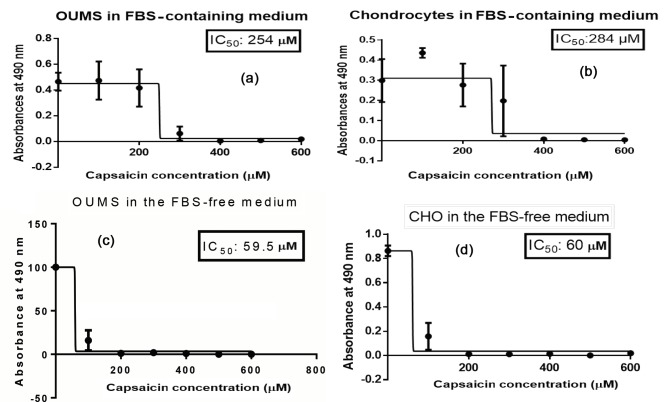
Absorbance at 490 nm versus capsaicin concentration (0–600 μM) graphs of (a) cancerous cells (OUMS) in FBS-containing medium, (b) normal cartilage cells (CHO) in FBS-containing medium, (c) OUMS in FBS-free medium, (d) CHO in FBS-free medium.

Cancerous cartilage cells presented an IC_50_ value of 254 µM (Figure 1a), which appeared to be lower than the one determined for healthy cells (284 µM) (Figure 1b) with no statistical significance. Moreover, an MTS assay was also performed in the absence of FBS for both cell types. The results were analyzed with GraphPad Prism 6.0 (Figures 1c and 1d). The IC_50_ values of 59,5 µM and 60 µM were calculated for the cancerous and normal cartilage cells grown in the absence of FBS, respectively. Despite the seemingly large difference between the cell types, two-way ANOVA resulted in no statistical significance.

**Figure 2:**
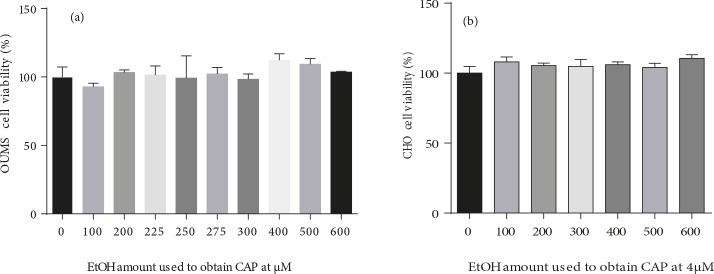
The effect of ethanol on (a) cancer cell (OUMS) and (b) normal chondrocyte cell (CHO) viabilities at the amounts used to obtain capsaicin concentrations varying between 0 and 600 μM. No statistically significant difference was detected between 0 and other concentrations (P > 0.05).

In these experiments, capsaicin was applied to the cells in a solution prepared with ethanol. In order to reveal the cytotoxicity of ethanol alone, we also applied ethanol in increasing amounts in each experiment. As a result, we observed that in the concentration range we investigated the effect of capsaicin, ethanol does not show cytotoxicity towards the examined cells (Figure 2).

### 3.2. Cyclin E protein almost disappeared when the cells were grown in FBS-deprived medium

**Figure 3:**
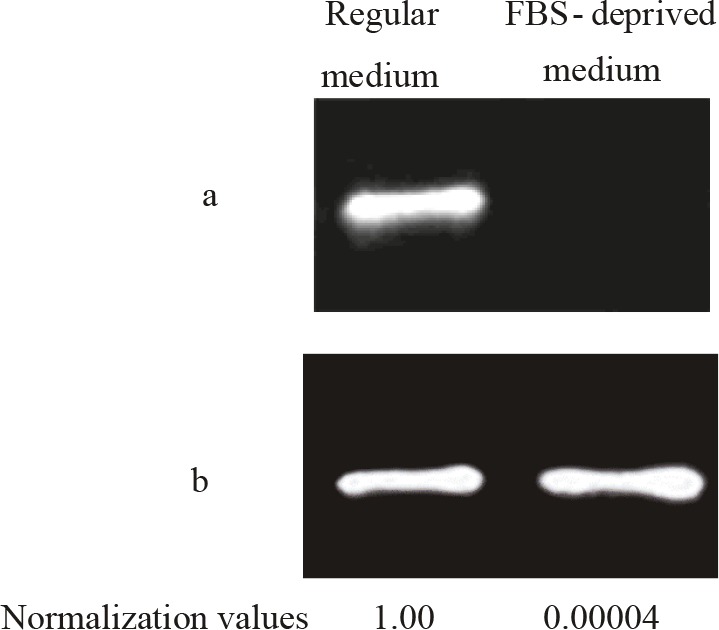
Cyclin E protein disappeared when OUMS cells were grown in FBS-deprived medium. Membrane image showing the bands for a) Cylin E b) GAPDH proteins of normal (CHO) and cancerous (OUMS) chondrocytes in regular and FBS-deprived medium. GAPDH was used for normalization purposes. Normalization was performed by dividing the band intensity of the targeted protein to that of GAPDH in the same well. Band ratio for regular medium was set as 1.00 and the other was calculated accordingly.

Cyclin E levels of the cells grown in FBS-containing and FBS-deprived media were also compared via western blotting, and it was observed that the cells decreased their cyclin E protein levels dramatically when grown in the medium with no FBS for 24 h. While Cyclin E relative band intensity reduced to 0.00004 for cancerous cells after 24 h in the medium without FBS compared to those in FBScontaining medium (1.00), the decline was calculated to be >99% for normal cells in the same conditions (Figure 3).

### 3.3. Caspase-3 protein and relative mRNA levels are not altered upon capsaicin exposure

**Figure 4:**
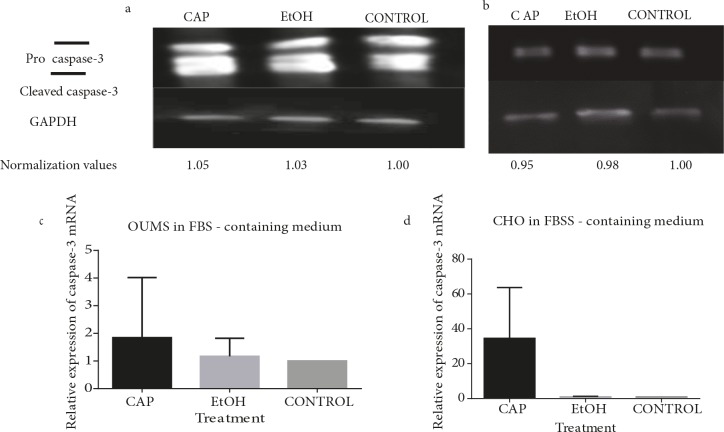
Capsaicin did not alter caspase-3 protein or mRNA levels in OUMS and CHO. Membrane images showing the bands for caspase-3 (upper) and GAPDH (lower) proteins in capsaicin-treated (CAP), ethanol-treated (EtOH), and untreated (CONTROL) (a) chondrosarcoma cells (OUMS) and (b) normal chondrocytes for 24 h. GAPDH was used for normalization purposes. Normalization was performed by dividing the band intensity of the targeted protein to that of GAPDH in the same well. The control group was set as 1.00 and the others were calculated accordingly. (c ) Analysis chart of RT-PCR results of chondrosarcoma cells (OUMS) grown in FBS-containing medium for caspase-3 mRNA. Despite a seemingly higher expression, the difference in mRNA levels was insignificant between capsaicin-treated (CAP), ethanol-treated (EtOH), and untreated (CONTROL) cells (P > 0.05). (d) Analysis chart of RTPCR results of normal cartilage cells (CHO) grown in FBS-containing medium for caspase-3 mRNA. Despite the seemingly higher expression, the difference in the mRNA levels was insignificant between capsaicin-treated (CAP), ethanol-treated (EtOH), and untreated (CONTROL) cells (P > 0.05).

In our study, neither cancerous nor normal cartilage cells displayed a change in their caspase-3 protein levels upon capsaicin exposure implying that the cell death observed in these populations occurred independently of caspases (Figures 4a and 4b). The fold change in capsaicin-treated, ethanol-treated, and untreated cancerous cells were 1.05, 1.03, and 1, respectively. For normal cells, however, these values were calculated to be 0.95, 0.98, and 1.

The mRNA levels of β-Actin control and caspase-3 were detected for chondrosarcoma and chondrocyte in real time. No cells presented a remarkable change in their caspase-3 mRNAs between capsaicin-treated, ethanoltreated, and untreated samples. Mean values and standard deviations of fold changes were calculated as 1.84 ± 2.17, 1.16 ± 0.65, 1.00 ± 0.00 and 34.53 ± 29.18, 1.04 ± 0.24, 1.00 ± 0.00 for capsaicin-treated, ethanol-treated, and untreated cells of OUMS and CHO, respectively. Although some increase or decrease was observed in individual experimental setups, the statistical analysis revealed no significance when all the values that were obtained from all experiments were evaluated together (P > 0.05) (Figure 4).

### 3.4. Cancerous cells migrated at a much slower rate with capsaicin

**Figure 5:**
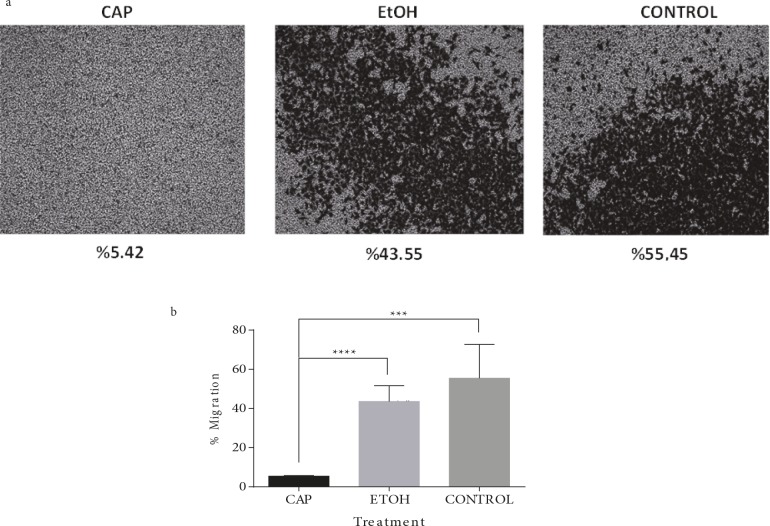
Capsaicin decreased the migration ability of OUMS cells grown in regular medium. (a) Representative pictures of migrated cancerous cells (OUMS) treated with capsaicin (CAP), ethanol (EtOH), or nothing (CONTROL) to the membrane surface touching the FBS-containing medium after 24 h. Percentages show the mean values calculated for the confluence of the cells in the pictures taken in replicated experiments. (b) Student’s t-test analysis of the values calculated for the pictures is represented in Figure 5a. The migration of capsaicin-treated (CAP) cells was significantly lower than that of both ethanol-treated (EtOH, ****P < 0.0001) and untreated (CONTROL, ***P = 0.0001) cells. The difference between EtOH and CONTROL, on the other hand, showed no statistical significance (P > 0.05).

The cells’ ability to move was evaluated in vitro with a migration assay. According to the results of the experiment, the ability of cancer cells to migrate appeared to be reduced by the treatment of capsaicin even though only the ethanol-treated cells became limited in this action in comparison to the untreated control. Each treatment was made in three repetitions and one representative picture for each condition was displayed in Figure 5a. The migration percentage values were calculated as 5.42 ± 0.37%, 43.55 ± 8.10%, and 55.45 ± 17.20% for capsaicin-treated, ethanoltreated, and untreated cancerous cells, respectively. When analyzed, the results showed that the capsaicin-treated cells were significantly slower in migration in comparison to their ethanol-treated and untreated counterparts (P = 0.0001 and P < 0.0001, respectively) (Figure 5b).

### 3.5. Cells’ ability to heal the wound dropped dramatically in capsaicin-containing environment

**Figure 6:**
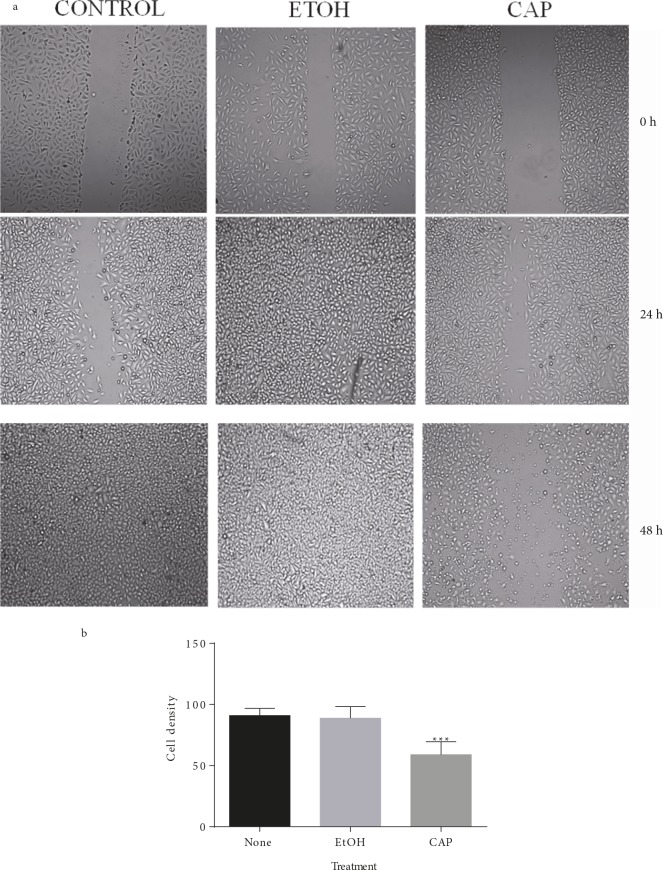
Capsaicin lowers the wound healing rate in OUMS grown in regular medium. (a) Representative pictures of migrated cancerous cells (OUMS) treated with capsaicin (CAP), ethanol (EtOH), or nothing (CONTROL) in FBS-containing medium at 0, 24, and 48 h of wound healing assay. Once fully confluent, a scratch was made through the cell layer of each well in a 6-well plate. Pictures were taken for each plate until the scratch was healed in at least one treatment. The changes in the wounded area were determined through the analysis of the pictures by ImageJ. (b) Student’s t-test analysis of the values calculated for the pictures is represented in Figure 6a. The migration of capsaicin-treated (CAP) cells was significantly lower than that of both ethanol-treated (EtOH) and untreated (CONTROL) cells (P < 0.01). The difference between EtOH and CONTROL, on the other hand, showed no statistical significance (P > 0.05).

**Figure 7:**
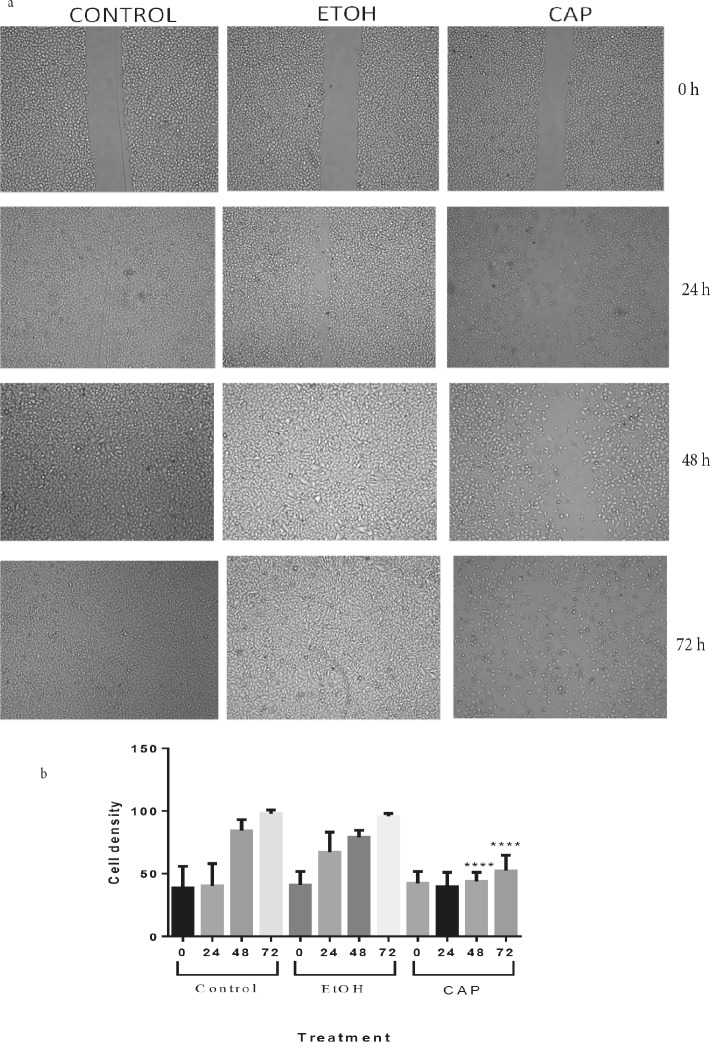
Capsaicin lowers the wound healing rate in OUMS grown in FBS-deprived medium. (a) Representative pictures of migrated cancerous cells (OUMS) treated with capsaicin (CAP), ethanol (EtOH), or nothing (CONTROL) in FBS-deprived medium at 0 and 72 h of wound healing assay. Once fully confluent, a scratch was made through the cell layer of each well in a 6-well plate. Pictures were taken for each plate until the scratch was healed in at least one treatment. The changes in the wounded area was determined through the analysis of the pictures by ImageJ. (b) Student’s t-test analysis of the values calculated for the pictures is represented in Figure 7a. The migration of capsaicin-treated (CAP) cells at 48 and 72 h was significantly lower than both ethanol- treated (EtOH) and untreated (CONTROL) cells (P < 0.001). The difference between EtOH and CONTROL at the same time points, on the other hand, showed no statistical significance (P > 0.05).

The assay was performed to understand whether capsaicin could effect the invasion of OUMS cells. The results showed that the invasion of OUMS cells was notably reduced by capsaicin. As it was visualized by the pictures presented in Figure 6a, treatment with capsaicin was able to attenuate the migratory ability of OUMS cells. At 48 h, the confluences of untreated and ethanol-treated cells were 90.89 ± 5.96 and 88.85 ± 9.41, respectively. Capsaicintreated cells, however, displayed a significantly lower level of confluence (58.92 ± 10.65). The data for this study were calculated and plotted with GraphPad Prism 6.0 (Figure 6b). In order to eliminate the criticism questioning whether the differences that were observed among the treatments were a result of weakened cell proliferation, an experiment was performed in the FBS-deprived medium since, as it was shown, the cells in the FBS-free medium lacked cyclin E and therefore were unable to move from the G1 phase to the S phase. The pictures were analyzed with ImageJ and the values were compared by GraphPad Prism 6.0 (Figure 7).

## 4. Discussion

The purpose of our study was to evaluate the potential of capsaicin to be used as a cancer treatment as well as understanding how its effectiveness will be regulated by reduced cyclin E levels. For this purpose, we used MTS Assay, western blotting, real-time PCR, wound healing, and migration assay.

We began our research by determining the IC_50_ values of the cells. In previous studies, numerous IC_50_ values were calculated for capsaicin on different cancer cell lines. For instance, in studies conducted on two different cell lines of colon cancer, HCT-116 and CaCo2, the IC_50_ values were given as 66.77 ± 10.78 µm and 163.70 ± 9.32 µm, respectively (Li et al., 2018). The IC_50_ values of the MTS assay with two cell lines (CEM/ADR 500 and CCRFCEM) of childhood T acute lymphoblastic leukemia, on the other hand, were found to be 125.85 ± 22.05 µm and 67.55 ± 6.29 µm, respectively (Li et al., 2018). In another study, the IC_50_ value of capsaicin in an osteosarcoma cell line was determined as 165.7 µm (Jin et al., 2016). The IC 50 we found in our experiments was 254 µm for cancerous cells, which appeared to be higher than those of most of the cancer types presented above. This observation was not unexpected when the aggressiveness and resistance of these cells to therapy are considered (Leddy and Holmes, 2014).

When the IC_50_ values of normal and cancerous cells grown in the FBS-containing medium were compared, it seemed that the value calculated for chondrocytes was roughly 11% higher than that of the cancerous cells, which might suggest that, if used at the right concentration, the substance may be able to eliminate cancerous cells without harming the normal cells. However, such a conclusion would be faulty because, despite the seemingly different IC_50_ values, the change in viability upon increasing concentrations of capsaicin turned out to be insignificant when two-way ANOVA analysis was applied to the data, implying that capsaicin was affecting both cell types similarly.

In the light of the results obtained through western blotting and RT-PCR performed for caspase-3, we would like to highlight that the death that was observed in both cell types did not show any sign of caspase-dependency, indicating that capsaicin is most likely to eliminate the cells through a pathway other than apoptosis. This result, albeit being partly unexpected due to the number of studies showing apoptotic effects of capsaicin on various cancer cell lines (Clark and Lee, 2016), was not unusual since there are many other natural products which eliminate cancer cells through nonapoptotic pathways and are still able to provide therapeutic potential because apoptosis is not the only type of programmed cell death to be used to eradicate tumors without surgery (Ye et al., 2018) .

Some readers might be surprised by the fact that we dissolved capsaicin in ethanol since DMSO is the most commonly used solvent in such studies (Wu et al., 2006; Zhang et al., 2008; Li et al., 2018) . We worked with ethanol-solved capsaicin because DMSO was shown to change cell behavior at lower concentrations than ethanol does (Timm et al., 2013) and in our preliminary trials, we observed that ethanol-solved-capsaicin was more potent against our cell lines than the DMSO-solved one (data not shown). The fact that using ethanol as the solvent increases the efficiency of capsaicin might be explained by the results of a research conducted by Mustafa and Ismael (2017). In the report they published, the authors stated that ethanol has the potential to induce a TRPV1-regulated response (Mustafa and Ismael, 2017). Considering that TRPV1 is the only known receptor of capsaicin, it is legitimate to assume that, when applied with ethanol, capsaicin might activate TRPV1 more than it would do without it, thereby generating an enhanced response thanks to the adding impact of ethanol on the same receptor. However, until it is evidenced by experimental data, the statement presented in the previous sentence remains as a speculation.

Growing cells in an FBS-deprived medium for 24 h lowered their cyclin E levels dramatically (P < 0.05). This dramatic decline of cyclin E levels without a drop in cell viability was read as a sign of G1 phase arrest of the cells because of the fact that cyclin E plays an essential role in G1-S phase transition through a cell division cycle (Teixeira and Reed, 2017) . Although we are aware that the decrease in cyclin E levels alone is not a real indication of G1 arrest because its levels are also low in the G2 and M phases of the cell cycle (Hochegger et al., 2008), it was not unreasonable to assume that this was the case, especially considering the healthy morphology and slow proliferation rate of the cells (data not shown) observed along with the lowered cyclin E levels. Nevertheless, in order to ensure that lowered FBS-deprived medium indeed arrested the cells in G1 phase, a flow-cytometry-based experiment had to be performed. The lack of this type of data remains as a limitation of our approach and prevents us from any cellarrest-related conclusion.

The reason why cyclin E reduction resulted in such observations, on the other hand, can be interpreted under the light of the recent literature. There are many reports where overexpression of cyclin E was related to the malignant behavior of certain cancers including lower survival rate in patients, genomic instability of the tumor cells, and a high cell proliferation rate (reviewed by Hwang and Clurman, 2005). These findings may imply that, with lower amounts of cyclin E, cell cycle process is disrupted, cells become less malignant and thus are more prone to the treatments that lead to cell death. However, in a more recent study with multiple myeloma cells, cyclin E expression levels of the cells were found not to be strictly related to their response to an apoptotic stimulus. In that report, the authors mentioned that some cells with high cyclin E expression profile were resistant to the stimuli while the others were rather sensitive to it, indicating a more complex system of regulation linked to the protein, which should be addressed in future studies (Josefsberg et al., 2012).

Regardless of the ambiguity about how the cell cycle was regulated by it, the FBS-deprived medium had a remarkable impact on capsaicin efficiency, since for both cell types, the IC_50_ values dropped dramatically when capsaicin was applied to the cells after they were cultured in the medium without FBS for 24 h (P < 0.0001). The specificity of the compound, however, did not appear to be affected by this application as two-way ANOVA analysis revealed no statistical significance between the cell types.

Although we cannot compare our results to previous reports due to the lack of studies of this kind, we can still discuss the observation in terms of the direct impact of FBS presence in the medium. In order to make such an evaluation correctly, we first need to consider the functions of serum in culture media. As it was reviewed by Bettger and McKeehan (1986) , serum in culture media detoxifies and stabilizes the factors required to maintain a favorable growth environment, provides hormone factors for cell proliferation; supplies essential nutrients, transport proteins, adherence and extension factors, trace elements, and promotes cell differentiation. Considering that serum supplements the medium with such important ingredients for cell growth and well-being, we may speculate that the absence of FBS might directly effect the drug response of a cell. Especially, deprivation of the factors that promote cell proliferation might be expected to increase the vulnerability of the cells against antiproliferative agents. Actually, a report indicating such a relation between FBS content and drug response was published by Fang et al. (2017).

Despite the fact that the cells grown in FBS-deprived medium reduced their cyclin E levels and IC_50_ values for capsaicin treatment, our preliminary RT-PCR results did not present any change in their caspase-3 levels (data not shown). These data may mean that the cell death pathway remained caspase-independent even though the mortality of the cells increased dramatically without FBS in the serum. We may, therefore, assume that whether a cell will die through caspase-dependent or caspase-independent pathway might be determined without the intervention of cyclin E protein.

As for wound healing and migration assays, we found that capsaicin reduced the motility of the cancerous cells dramatically in both assays. This finding was similar to the observations in the literature for other cancer types such as melanoma and breast cancer (Shin et al., 2008; Li and Yuan, 2017). In order to address the potential criticism that would suggest that what we observed in the wound healing assay was a natural outcome of fastergrowing cells and therefore could not be interpreted as the inhibition of motility upon capsaicin exposure, we repeated our experiments in the FBS-deprived medium. Since the cells were not expected to grow fast enough to heal the wound in 24 h, we believe that the difference we evidenced for capsaicin-treated and untreated cells in that experiment supported the assumption that capsaicin significantly reduced the migrating ability of OUMS. The results of this experiment were backed even further with the migration assay we performed. On the other hand, the observations we made for the migration of capsaicin-treated cells matched with the literature only partially. In a paper by Lee et al. (2014), the migrations of cholangiocarcinoma cells were found to be slowed down by the presence of capsaicin in vitro, and down-regulation of matrix metalloproteinase-9 through AMPK–NF-κB pathway was given as its possible mechanism. Bitencourt et al. (2014) showed that capsaicin reduced the cell migration significantly in hepatic stellate cells. Conversely to these reports, Liu et al. (2012) demonstrated that low concentrations of capsaicin upregulated tNOX (tumorassociated NADH oxidase) expression in HCT116 human colon carcinoma cells and enhanced the migration of the cells in vitro and in vivo.

The fact that a single cell line and one primary cell population were used in the study is the biggest limitation of our approach. In order to have a better understanding about the actual impact of capsaicin on chondrosarcoma and chondrocytes, various cell lines generated from all grades of tumors and a higher number of primary cell populations that are obtained from individuals with different sexes, ages, and races must be included in future studies.

To our knowledge, the results that are presented here are among the firsts on the effect of capsaicin on a chondrosarcoma cell line. Apart from that, we also investigated the impact of the substance on normal cells of the same tissue to scrutinize its potential to be used as a therapeutic agent more realistically. Although we experimentally showed that capsaicin had a cytotoxic potential against chondrosarcoma cells and was able to reduce their migratory and invasive capacities, the fact that normal chondrocytes were also almost equally eliminated by its exposure seemed to annihilate its potential use in therapy.

## Acknowledgment

This study was supported by Kahramanmaraş Sütçü İmam University Scientific Research Project Unit with the project number of 2017 / 4-27 D.
